# Management of the axilla after neoadjuvant chemotherapy in breast cancer patients

**DOI:** 10.1093/bjs/znab137

**Published:** 2021-05-16

**Authors:** M R Boland, Z Al-Hilli

**Affiliations:** 1 Department of Surgery, Royal College of Surgeons in Ireland, Dublin, Ireland; 2 Department of General Surgery, Digestive Diseases and Surgery Institute, Cleveland Clinic, Cleveland, Ohio, USA

## Introduction

The use of neoadjuvant chemotherapy (NACT) in treating newly diagnosed breast cancer patients has increased significantly in recent years. Traditionally NACT was used in locally advanced and inflammatory breast cancer and for patients with large operable cancers who desired breast-conserving surgery. In recent years, focus has shifted towards the role of NACT in de-escalating axillary surgery and associated morbidity, as well as assessing and guiding systemic therapy based on treatment response in the breast and axilla. In particular, patients with specific breast cancer subtypes, namely triple negative breast cancer (TNBC) or hormone receptor negative/human epidermal growth factor receptor (HER2) positive cancers, can achieve complete pathological response (pCR) approaching 70 per cent in the breast and axilla. It is likely that many of these patients would benefit from minimally invasive approaches to axillary surgery. The aim of this review is to provide a current update and future direction regarding the management of the axilla in breast cancer patients treated with NACT.

### Current evidence for management of nodal disease

Some have advocated the use of sentinel node biopsy to evaluate the axilla before NACT, but studies have shown that sentinel node biopsy after NACT is of higher prognostic value^1^. Patients who are clinically node negative at diagnosis should receive NACT and undergo sentinel node biopsy as standard axillary assessment after treatment.

Recent evidence has also suggested that patients with clinically node-positive disease at diagnosis with a clinical and radiological response to NACT are candidates for sentinel node biopsy after NACT^2^. Publication of three large RCTs, ACOSOG Z1071, SENTINA and SN FNAC, demonstrated that, in patients who were initially node positive and received NACT, an acceptable false-negative rate of 10 per cent was achievable by removing at least three nodes and using dual tracer mapping of the sentinel lymph nodes^3–5^. Currently, patients found to have a positive sentinel node biopsy after NACT still require an axillary lymph node dissection (ALND).

## Safety of sentinel node biopsy after neoadjuvant chemotherapy

Another method to reduce the false-negative rate of sentinel node biopsy after NACT in patients who were initially node positive is concurrently to remove the abnormal nodes identified at the time of initial diagnosis in addition to dual mapping and removal of the sentinel lymph nodes. Targeted axillary dissection was first described by Caudle and colleagues in 2016^6^ and involves radiological clipping of the abnormal node at diagnosis and subsequent removal of the sentinel nodes as well as the abnormal node identified preoperatively by localization of the clipped node. This technique can result in a reduction in the axillary false-negative rate from 10 to 2.4 per cent. In about 75 per cent of patients the clipped node will be one of the sentinel nodes. Other techniques described involve placement of a radioactive seed into the abnormal node at diagnosis, which is then identified at surgery after NACT using a radioisotope gamma probe and subsequently removed, again in addition to the sentinel node. This technique, described by a group from the Netherlands Cancer Institute, can reduce the false-negative rate to 7 per cent, making it an acceptable alternative^7^. More recently the use of superparamagnetic iron oxide tracer to identify sentinel nodes has also been examined both in Europe and the USA with promising results^8^.

## Future directions

Two ongoing trials are addressing the local management of the axilla in patients with clinically node positive disease at presentation. These will likely add significantly to the evolving management of the axilla after NACT. The NRG Oncology/NSABP B-51/RTOG 1304 study is examining the need for regional nodal irradiation in patients who were initially node positive and converted to node negative after NACT, and this trial has invasive breast cancer recurrence free interval as the primary outcome. Meanwhile, the Alliance for Clinical Trials in Oncology A011202 examines the need for ALND in patients found to have a low positive axillary nodal burden after NACT. In this study patients with a positive sentinel node biopsy after NACT are randomised to completion ALND and regional nodal irradiation or to regional nodal irradiation only, with the primary outcome again being invasive breast cancer recurrence free interval. Both studies are likely to reduce the need for ALND or nodal irradiation after NACT in these select populations.

## Tumour biology

It is also likely that as more is understood about the biology of each patient’s breast cancer, tailored and individualised approaches to surgical management of the axilla will be adopted, with a focus on maximising outcomes whilst minimising morbidity. Within the breast, a number of studies have attempted to identify patients who achieve a pCR and thus may not require surgery at all^9^. Whilst this has yet to translate into common clinical practice, it is likely that a similar pattern may be observed in the axilla, again minimising and even removing the morbidity associated with both sentinel node biopsy and ALND, such as seroma formation and lymphedema. Although receptor subtypes including oestrogen/progesterone (ER/PR) and Her2 positivity are still most commonly used when deciding on NACT, there is now strong evidence that multigene molecular signatures likely will be more commonly used to identify patients most likely to achieve a pCR^10^. Patients found to be ER positive generally have lower rates of pCR compared to those with HER2 positive or triple negative breast cancer. Using a molecular signature in this setting can identify patients in the ER positive group who are more likely to achieve pCR and avoid ALND. Equally, identifying those with a signature indicating chemoresistance may be more suitable to primary surgery.

## Conclusion

NACT is increasingly utilized in patients with clinically node positive disease in order to down-stage the axilla and de-escalate the extent of surgery, thereby reducing risks related to surgery. Local management, i.e., surgery and radiation therapy, continues to evolve as the impact of treatment on patient outcomes is better understood. A tailored approach, taking into consideration hormone receptor subtype/tumour biology and response to NACT is likely to continue to evolve and impact local management.

*Disclosure*. The authors declare no conflicts of interest.
